# Morimoto Y, Nishida C, Tomonaga T, et al. Lung disorders induced by respirable organic chemicals. J Occup Health. 2021; 63:e12240. https://doi.org/10.1002/1348‐9585.12240

**DOI:** 10.1002/1348-9585.12384

**Published:** 2023-01-19

The publisher would like to draw the reader's attention to an error in the article.

Table 1 has been published incorrectly in this article.

**TABLE 1:** Work‐related asthmas and their classifications

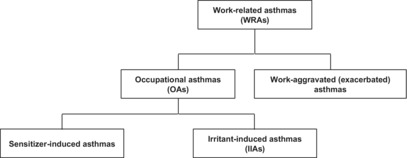

• Work‐related asthmas (WRAs) mean those that are related to occupations.

• Occupational asthma (OA) means those that are related to occupations and caused by allergens existing in the workplace.

• Sensitizer‐induced asthmas mean those that are associated with an immunological and allergic mechanism.

• Irritant‐induced asthmas (IIAs) mean those that occur due to aspiration of a large quantity of an irritant at the workplace.

• Work‐aggravated (exacerbated) asthmas mean those that are pre‐existent and aggravated by gas, cool air, or dust aspirated at the workplace.

The correct Table 1 is shown below.

The publisher apologizes for this error and any confusion it may have caused.

